# A systematic review assessing the effectiveness of COVID-19 mRNA vaccines in chronic kidney disease (CKD) individuals

**DOI:** 10.12688/f1000research.122820.1

**Published:** 2022-08-08

**Authors:** Soniya A. Malik, Kavindiya Modarage, Paraskevi Goggolidou

**Affiliations:** 1 Research Institute in Healthcare Science, Faculty of Science and Engineering, University of Wolverhampton, Wulfruna Street, Wolverhampton, WV1 1LY, UK

**Keywords:** Chronic Kidney Disease, COVID-19, Dialysis, IgAN, mRNA Vaccines, Kidney Transplant

## Abstract

**Background: **SARS-CoV-2 is a coronavirus that has rapidly spread across the world with a detrimental effect on the global population. Several reports have highlighted an increased mortality rate and a higher severity of COVID-19 infection in chronic kidney disease (CKD) individuals. Upon the development of various SARS-CoV-2 vaccines, mRNA vaccines including BNT162b2 and mRNA-1273 were deemed safe, with a high efficacy in preventing COVID-19 in the general population. This review investigates whether  SARS-CoV-2 mRNA vaccines are as effective in triggering an immune response in Dialysis Patients (DPs) and Kidney Transplant Recipients (KTRs) and if a third dose is required in this  population.

**Methods: **A systematic search employing the PRISMA criteria was conducted in several major databases, with the data being extracted from publications for the period January 2021 to May 2022 (PROSPERO: CRD42022338514, June 15, 2022).

**Results: **80 studies were included in this analysis with a total cohort number of 15,059 participants. Overall, 85.29% (OR = 17.08, 95% CI = 15.84-18.42, *I ^2^* = 98%) and 41.06% (OR = 0.52, 95% CI = 0.48-0.5, *I ^2^* = 95%) of DPs and KTRs included in this review showed positive seroconversion after two doses of either mRNA vaccine, respectively. A total 76% (OR = 6.53, 95% CI = 5.63-7.5, *I ^2^* = 96%) of the cohort given a third dose of an mRNA vaccine demonstrated positive seroconversion, with 61.86% (OR = 2.31, 95% CI = 1.95-2.75 *I ^2^* = 95%) of the cohort that was assessed for a cellular response displaying a positive response.

**Conclusions: **This data emphasises a reduced incidence of a positive immune response in DPs and KTRs compared to healthy controls, albeit a better response in DPs than when compared to KTRs alone was observed. A third dose  appears to increase the occurrence of an immune response in the overall DP/KTR cohort.

## Introduction

In December 2019, an outbreak of atypical respiratory disease was reported in Wuhan, Hubei Province, China ( Kumar *et al.,* 2021). This was later confirmed to be caused by a novel coronavirus, formally recognised by the World Health Organisation (WHO) as Severe Acute Respiratory Syndrome Coronavirus 2 (SARS-CoV-2) or Coronavirus Disease-2019 (COVID-19) ( Tsang *et al.,* 2021). SARS-CoV-2 rapidly spread across the globe, with WHO declaring it a Public Health Emergency of International Concern (PHEIC) on January 30, 2020 ( Cucinotta and Vanelli, 2020). Shortly after, COVID-19 was declared a pandemic by WHO on March 11, 2020, with current data from WHO reporting a cumulative total number of 521,920,560 cases and 6,274,323 total deaths as of May 23, 2022 ( Cucinotta and Vanelli, 2020; *WHO Coronavirus (COVID-19) Dashboard*).

SARS-CoV-2 is a β-coronavirus; coronaviruses consist of enveloped, positive-sense, single-stranded RNA, and non-structural, structural and accessory proteins ( Chan *et al.,* 2020; Yadav *et al.,* 2021). Each of these proteins play a crucial role in the process of viral-host invasion. In the initial stages of infection, the spike (S) protein is a crucial factor for viral entry. The S protein is composed of two functional subunits, S1 and S2. The S1 subunit of the S protein in SARS-CoV-2 is responsible for binding to the cellular ligand on Angiotensin Converting Enzyme 2 (ACE2) receptor on the host cell surface ( Tsang *et al.,* 2021; Yuki, Fujiogi and Koutsogiannaki, 2020; Hoffmann *et al.,* 2020; Li *et al.,* 2003). Following this interaction, transmembrane protease serine 2 (TMPRSS2) plays a crucial role in priming the S1/S2 cleavage site, leading to the stabilisation and subsequent cleavage of the S2 subunit ( Hoffmann *et al.,* 2020; Tsang *et al.,* 2021; Belouzard, Chu and Whittaker, 2009). This presumably activates the S protein, facilitating the viral and host cell membrane fusion ( Belouzard, Chu and Whittaker, 2009; Hoffmann *et al.,* 2020; Ou *et al.,* 2020). Upon cell entry, the replicase complex begins the process of transcription and translation, leading to the synthesis of new viral RNA and proteins. At this stage, the nucleocapsid (N) protein will begin virion assembly and bind new genomic RNA, whilst the S, envelope (E) and membrane (M) structural proteins, which comprise trafficking signal sequences, translocate to the endoplasmic reticulum ( Chang *et al.,* 2005; Chen, Liu and Guo, 2020; Yadav *et al.,* 2021; Tsang *et al.,* 2021). The M protein plays a crucial role in shaping virions and binding to the nucleocapsid, whilst the E protein then plays a direct role in viral pathogenesis, assembly and eventual release out of the cell via exocytosis ( Neuman *et al.,* 2011; Chen, Liu and Guo, 2020; Yadav *et al.,* 2021).

The initial point of entry for SARS-CoV-2 is the respiratory tract, where the virus will enter and bind to nasal epithelial cells in the upper respiratory tract, via ACE2 ( Sungnak *et al.,* 2020). ACE2 is expressed in several organs including the heart, lungs and intestine ( Tsang *et al.,* 2021; Zou *et al.,* 2020). Importantly, the highest levels of expression of ACE2 can be found in the kidneys, especially in renal tubular cells ( Hamming *et al.,* 2004). However, it has been reported that ACE2 expression can vary within kidney disease and renal transplant patients, with a reduced level of ACE2 expression also reported in diabetic nephropathy ( Tikellis *et al.,* 2003; Lely *et al.,* 2004). Upon investigation, one *in vivo* study highlighted that chronic pharmacologic inhibition of ACE2 could worsen glomerular injury ( Soler *et al.,* 2007). Kidney cells also express TMPRSSs, a co-receptor to viral entry ( Chen *et al.,* 2021). Since the kidney is a target organ for SARS-CoV-2, viral invasion and consequent replication in kidney cells have the potential to trigger a cytokine storm, initiating possible organ damage in patients ( Jin *et al.,* 2020). Population studies revealed that older patients were more prone to a severe clinical outcome alongside those who were clinically vulnerable and suffered from conditions including but not limited to diabetes, cardiovascular disease (CVD) and chronic kidney disease (CKD) ( Hu and Wang, 2021; Gansevoort and Hilbrands, 2020).

Developing research has quickly recognised that CKD patients are more susceptible and prone to developing severe outcomes due to SARS-CoV-2 infection. High mortality risk was associated with CKD for patients with eGFR <30 mL/min/1.73 m ^2^ (adjusted hazard ratio (aHR) 2.52), dialysis patients (aHR 3.69) and transplant patients (aHR 3.53), constituting some of the top risk categories in this study ( Williamson *et al.,* 2020). Similar to this, the 28-day mortality was 20.0% (95% confidence interval (CI) 18.7% - 21.4%) and 19.9% (17.5% - 22.5%) in 3285 dialysis patients (DPs) and 1013 kidney transplant recipients (KTRs), respectively ( Jager *et al.,* 2020). Another meta-analysis established a higher mortality rate in CKD patients with COVID-19 infection compared to those without COVID-19 infection (pooled OR 5.81 (95% CI 3.78 – 8.94)) ( Cai *et al.,* 2021).

Nearing the end of 2021, several SARS-CoV-2 vaccines have emerged for use with authorisation across the globe. According to WHO, as of May 17, 2022, a total of 12,186,798,032 vaccine doses have been administered ( *WHO Coronavirus (COVID-19) Dashboard*). Despite several studies investigating the safety and efficacy of vaccinations, there is no conclusive evidence as to whether the SARS-CoV-2 vaccines provide the same level of immunity in CKD patients. Overall, it has been deemed safe to use the mRNA vaccines BNT162b2 (Pfizer-BioNTech) and mRNA-1273 (Moderna) in immunocompromised patients, including CKD patients ( Windpessl *et al.,* 2021). Clinical trials have detected a higher level of efficacy in mRNA vaccines compared to viral-vectored vaccines (95% for BNT162b2, 94.1% for mRNA-1273, 70.4% for ChAdOx1 nCoV-19) ( Baden *et al.,* 2021; Polack *et al.,* 2020; Voysey *et al.,* 2021). All three vaccines have got the ability to induce a strong S-specific antibody response followed by T-cell immunity after two parenteral injections ( Folegatti *et al.,* 2020; Jackson *et al.,* 2020). However, since characteristics such as kidney disease, and dialysis treatment/transplantation could affect vaccination efficacy, this study will focus on investigating the evidence on whether the SARS-CoV-2 mRNA vaccines can indeed provide effective immunity to CKD individuals and if further action is required.

## Methods

### Search strategy and eligibility criteria

This systematic review was conducted in accordance with Preferred Reporting Items for Systematic Reviews and Meta-Analyses (PRISMA) guidelines, published in 2020. See *Reporting guidelines* ( Goggolidou, 2022) for the completed checklist. The study was registered in PROSPERO: CRD42022338514, on June 15, 2022 ( Page *et al.,* 2021). A preliminary search was first conducted on PubMed for publications detailing the efficacy of SARS-CoV-2 vaccination in CKD patients to validate the research question. This was followed by a systematic search by two independent reviewers which was last conducted on May 23, 2022 on PubMed, Google Scholar, Semantic Scholar, CORE, Science Open and BioMed Central using search terms and phrases of ‘efficacy of COVID-19 vaccine’, ‘immunogenicity of COVID-19 vaccine’, ‘chronic kidney disease’ and ‘COVID-19 booster’. Filters were applied whereby only studies published between January 2021 and May 2022 were included, with unpublished studies i.e. pre-prints also being included in this analysis. Inclusion and exclusion criteria were defined to ensure all relevant studies were identified. Only studies with an adult human cohort of any sex and published in any country were included. Patients under the age of 18 were excluded from this analysis. As well as this, studies or trials that specify the vaccine type including BNT162b2 and mRNA-1273 vaccinations were only included. Patients who received any other form of COVID-19 vaccine were excluded, since there was limited information available. The studies must have also mentioned at least the treatment regimen that patients were undertaking including dialysis or transplantation. If this was unclear, studies were excluded. Studies were grouped into categories according to the treatment regimen patients undertook and the vaccine that patients were given, including the number of doses. As well as this, studies were separated according to the type of analysis that was undertaken i.e., seroconversion and/or cellular analysis.

### Data extraction

**Figure 1.**
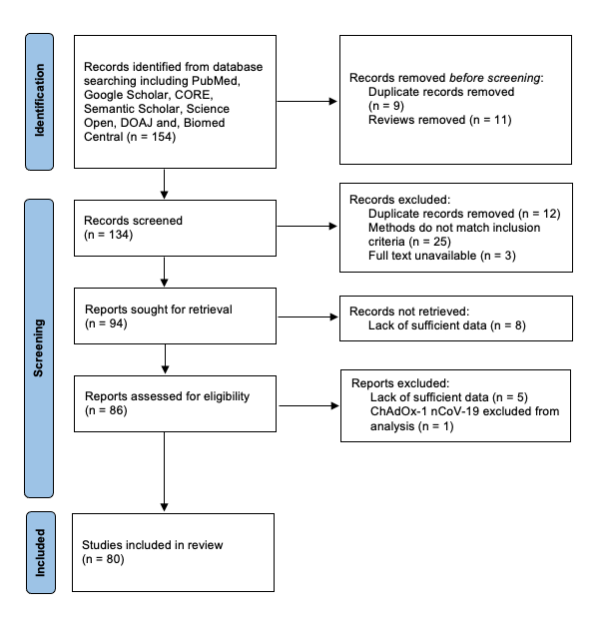
Preferred Reporting Items for Systematic Reviews and Meta-Analyses (PRISMA) statement demonstrating the different phases of study selection of the systematic review.

Studies were grouped together as described above with two independent reviewers assessing whether they met the inclusion criteria (S.A.M, K.M). Studies were assessed and data was collected via Microsoft Excel 2016 by the same reviewers. The information extracted included sample size and sample groups, treatment regimen, number of doses, whether there is a history of COVID-19 and how long after vaccination any assessments took place. The effects and responses to the vaccination including humoral and cellular immunity responses after the second and third doses were also reviewed (the PRISMA flow diagram is given as Figure 1). Any disputes were overlooked by P. G. Studies with missing or unclear data were removed from the analysis. The risk of bias and heterogeneity were assessed with the Cochrane’s Q test and I ^2^ test using RevMan (version 5.4) by two independent reviewers (S.A.M, K.M).

### Data analysis

Two independent reviewers assessed studies for eligibility for each synthesis using Microsoft Excel 2016. Studies were grouped in accordance with data availability before being included in the synthesis, with those with missing data being excluded from analysis. Funnel plots, forest plots and statistical analysis were performed using RevMan (version 5.4). Odds ratios (OR) and 95% confidence intervals (CI) were calculated to evaluate the seroconversion and cellular response in DPs and KTRs after two and/or three doses of the BNT162b2 and mRNA-1273 vaccinations. The seroconversion is a measure of antibody response to SARS-CoV-2-specific antibodies including IgG, IgA, and IgM antibodies. The cellular response incorporated the number of patients that developed a T-cell response across the studies included in this analysis, including CD4 ^+^ and CD8 ^+^ T cells. The population was divided into subgroups i.e., dialysis patients and transplant recipients; two dose and three dose groups; seroconversion and cellular response group. A pooled 95% CI not including 1 for the OR was considered as statistically significant. Risk of bias/heterogeneity was assessed using RevMan (version 5.4) with the Cochrane’s Q test and I ^2^ test and presented in funnel and forest plots. If a P value of < 0.1 and/or I ^2^ was > 50%, heterogeneity was determined as significant.

## Results

A total of 80 studies (76 published, 4 preprints) were included in this work, with an overall cohort number of 15,059 participants. Of these, 11,509 participants were recipients of the BNT162b2 vaccine, with 4,263 being KTRs and 7,246 participants being DPs. Separately, a total of 3,550 participants were vaccinated with the mRNA-1273 vaccine. Of these, 826 participants were KTR, and 2,724 participants were DP. Finally, 292 participants had been given the ChAdOx-1 nCoV-19 vaccination, with 11 participants being KTR and 281 participants being DP. Due to the lack of studies for ChAdOx-1 nCoV-19 vaccination in this population, this data was excluded from analysis and one study was ruled out ( Meshram *et al.,* 2021). Seroconversion and cellular response were assessed to investigate whether the mRNA vaccines were able to trigger a response in immunocompromised patients; to determine if a particular vaccine was more appropriate for DPs or KTRs; and to determine the ability of a third dose in inducing an antibody or cellular response in this population type.

**Figure 2.**
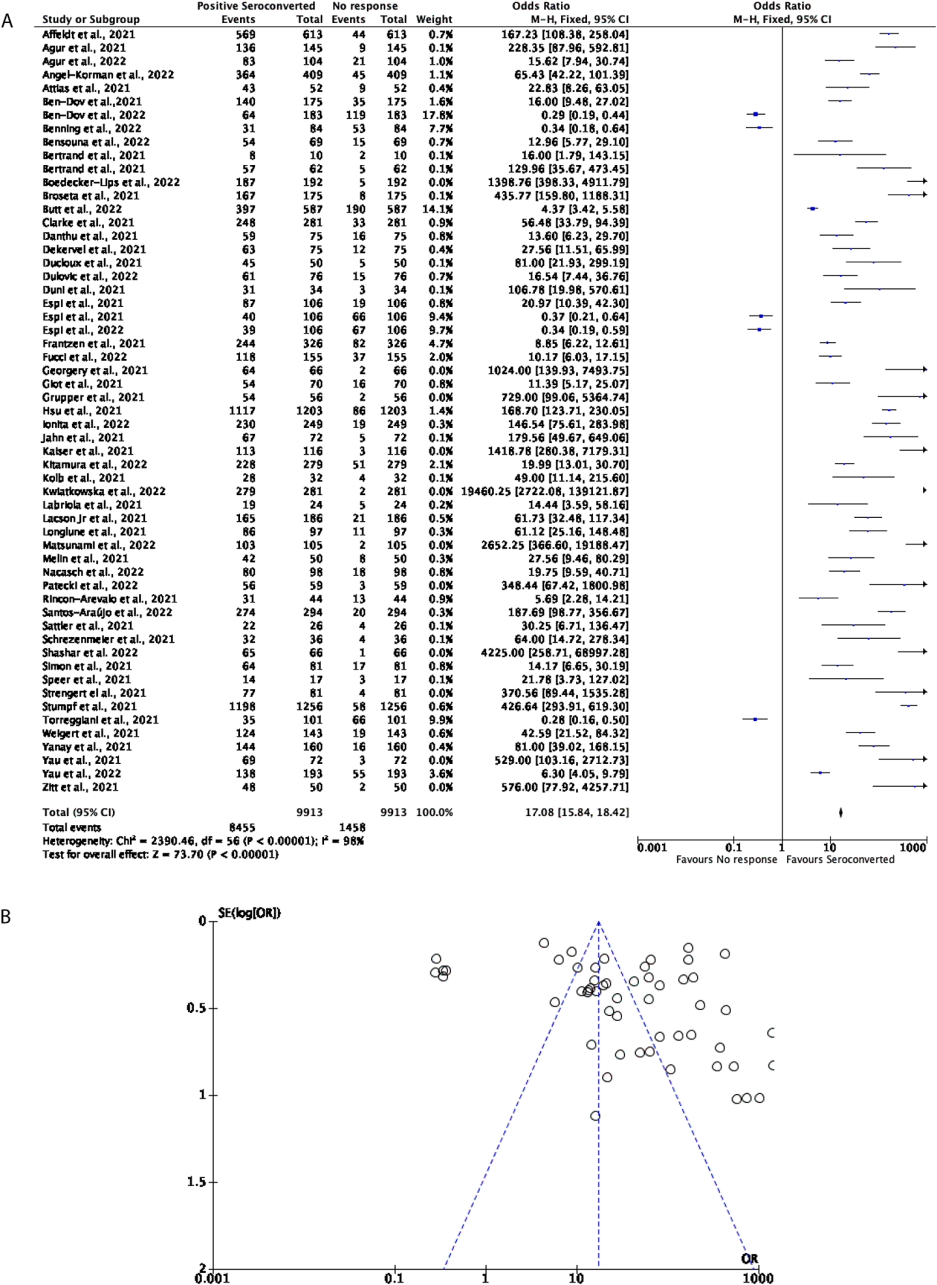
A total of 85% of Dialysis Patients (DPs) seroconvert after receiving two doses of a COVID-19 mRNA vaccine. (A) Forest plot comparison of the number of DPs who seroconverted or had no response after two doses of an mRNA vaccination (OR = 17.08, 95% CI = 15.84-18.42, *P* < 0.00001, *I ^2^* = 98%). (B) Funnel plot evaluating heterogeneity when comparing seroconversion response in DPs after two doses of an mRNA vaccine. Significant heterogeneity was observed ( *P* < 0.00001, *I ^2^* = 98%).

57 studies with a total of 9,913 participants were included to determine how effective two doses of the mRNA vaccines, including BNT162b2 and mRNA-1273, were in DPs. Overall, the mRNA vaccines prove to be effective in inducing seroconversion, with around 85.29% of participants positively seroconverting. All but five studies in this cohort favoured positive seroconversion with the remaining 14.71% of participants favouring no seroconversion response (OR = 17.08, 95% CI = 15.84-18.42, Figure 2A). Significant heterogeneity was observed ( *P* < 0.00001, *I* ^2^ = 98%, Figure 2B).

**Figure 3.**
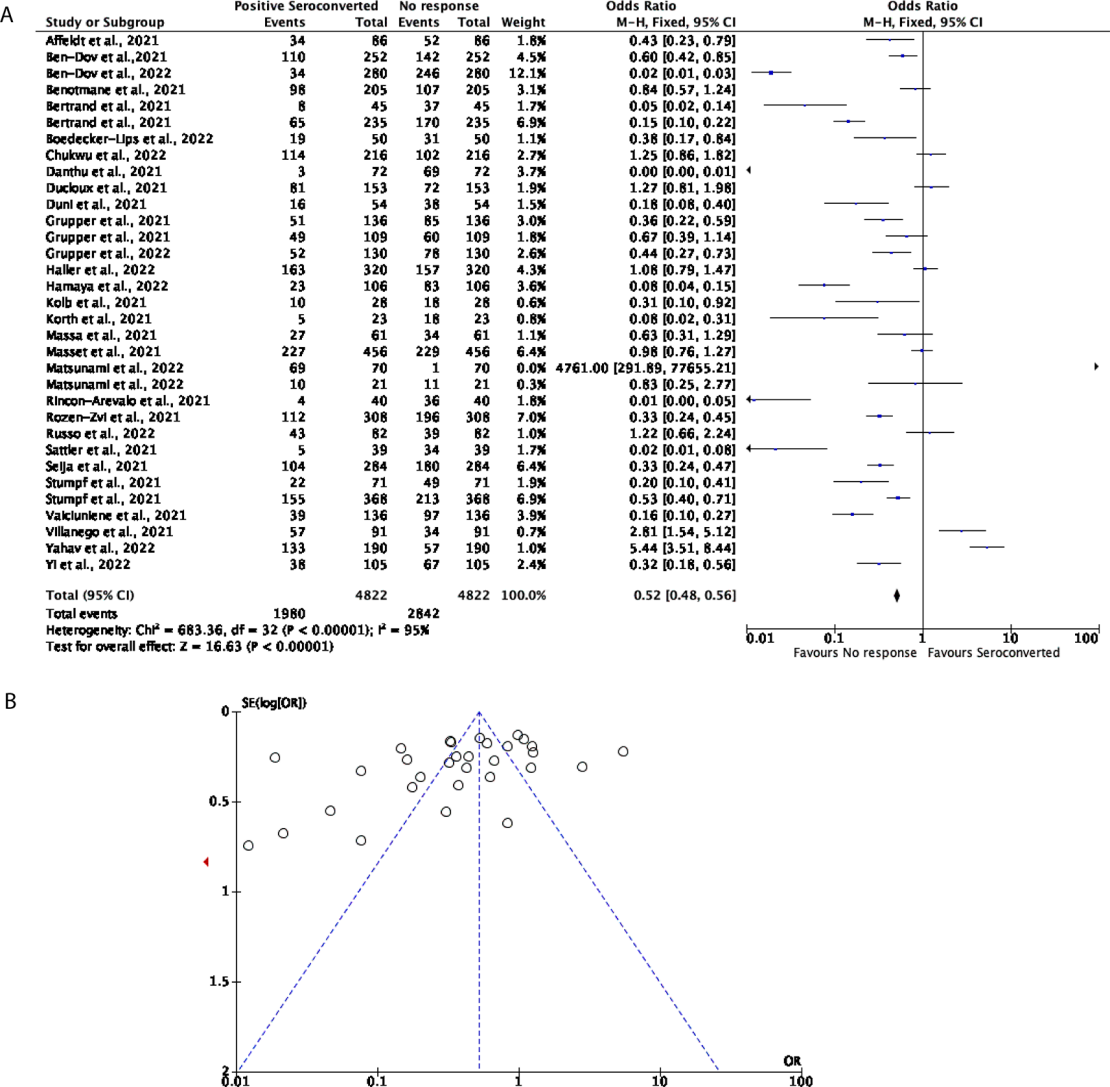
Only 41% of Kidney Transplant Recipients (KTRs) seroconvert after receiving a COVID-19 mRNA vaccine. (A) Forest plot comparison of the number of KTRs who seroconverted or had no response after two doses of an mRNA vaccination (OR = 0.52, 95% CI = 0.48-0.56, *P* <0.00001, *I ^2^* = 95%). (B) Funnel plot evaluating heterogeneity when comparing seroconversion response in KTRs after two doses of an mRNA vaccine. Significant heterogeneity was observed ( *P* <0.00001, *I ^2^* = 95%).

Comparatively, 33 studies with a total of 4,822 participants were included to determine the effectiveness of two doses of the mRNA vaccines including BNT162b2 and mRNA-1273 in KTRs. Compared to the seroconversion rate in DPs, the mRNA vaccines were not able to effectively trigger a positive seroconversion in the KTR population. 41.06% of participants displayed positive seroconversion, with the remaining 58.94% of participants showing no response (OR = 0.52, 95% CI = 0.48-0.56, Figure 3A). Significant heterogeneity was observed ( *P* < 0.00001, *I* ^2^ = 95%, Figure 3B).

**Figure 4.**
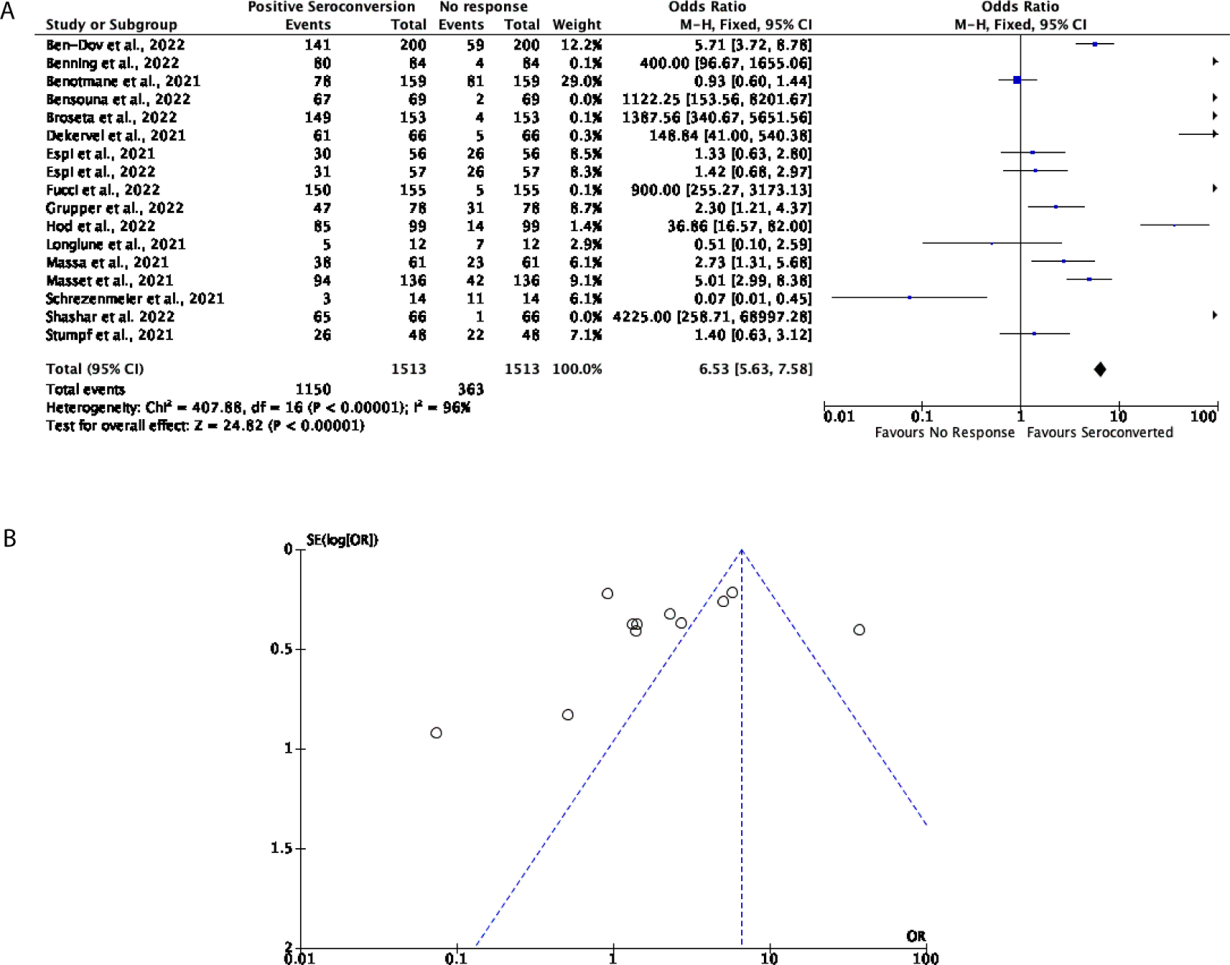
After three doses of a COVID-19 mRNA vaccine, 76% of Dialysis Patients (DPs) and Kidney Transplant Patients (KTRs) seroconverted. (A) Forest plot comparison of the number of DPs and KTRs who positively seroconverted after three doses of an mRNA vaccine (OR = 6.53, 95% CI = 5.63-7.58, *P < 0.00001, I ^2^* = 96%). (B) Funnel plot evaluating heterogeneity when comparing seroconversion in DPs and KTRs after three doses of an mRNA vaccine. Significant heterogeneity was observed ( *P < 0.00001, I ^2^* = 96%).

Aside from this, 17 studies with a total of 1,513 participants were included to determine the effectiveness of three doses of the mRNA vaccines, consisting of either the BNT162b2 or the mRNA-1273 in both DPs and KTRs. Of the participants that were included in this assessment, 76% displayed seroconversion after three doses of an mRNA vaccine, with 24% of participants showing no response; multiple studies included in this analysis demonstrate a strong, positive seroconversion after a third dose of an mRNA vaccine (OR = 6.53, 95% CI = 5.63-7.58, Figure 4A). Significant heterogeneity was however observed ( *P* < 0.00001, *I* ^2^ = 96%, Figure 4B).

**Figure 5.**
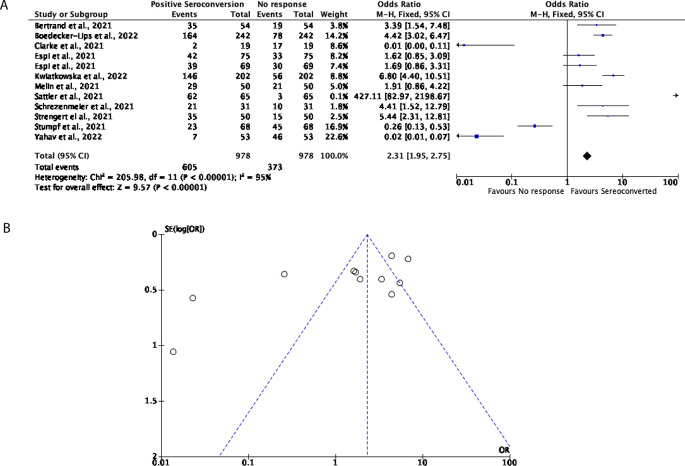
A total of 62% of Dialysis Patients (DPs) and Kidney Transplant Recipients (KTRs) elicited an effective cellular response following two doses of a COVID-19 mRNA vaccine. (A) Forest plot comparison of the number of DPs and KTRs who developed a cellular response after two doses of an mRNA vaccine (OR = 2.31, 95% CI = 1.95-2.75, *P* < 0.00001, *I ^2^* = 95%). (B) Funnel plot evaluating heterogeneity when comparing cellular response in DPs and KTRs after two doses of an mRNA vaccine. Significant heterogeneity was observed ( *P* < 0.00001, *I ^2^* = 95%).

Further to this, 12 studies with a total of 978 participants were included for this analysis to determine how many DPs and KTRs developed a cellular response after being vaccinated with two doses of either the BNT162b2 or mRNA-1273 vaccines. Overall, in this cohort, 61.86% of participants favoured an effective cellular response with a further 38.14% of participants showing no cellular response (OR = 2.31, 95% CI = 1.95-2.75) ( Figure 5A), nevertheless significant heterogeneity was observed ( *P* < 0.00001, *I* ^2^ = 95%; Figure 5B).

It has also been reported that IgAN or other kidney diseases could be triggered as a response to the COVID-19 mRNA vaccinations in certain populations ( Wisnewski, Campillo Luna and Redlich, 2021). IgA nephropathy (IgAN) is an autoimmune kidney disease characterised by the deposition of IgA in the glomerulus, which causes inflammation and damage in the kidney ( Selvaskandan *et al.,* 2019). We found five studies where it was reported that nine patients were diagnosed with IgAN or presented with hallmarks of IgAN, such as gross haematuria after having either the BNT162b2 or mRNA-1273 vaccine administered ( Table 1). Of these, six patients had previously diagnosed IgAN, with a further two patients having no significant medical history besides gestational diabetes and hyperlipidaemia. The final patient also had no significant past medical history. Most of these individuals were female with an average age of ~42 years old, with one study not specifying the age of the female and instead, using the description ‘older woman’ ( Perrin *et al.,* 2021; Rahim, Lin and Wang, 2021; Tan *et al.,* 2021; Sacker, Kung and Andeen, 2021; Negrea and Rovin, 2021). One case study reported the IgAN phenotype in a 22-year-old male ( Perrin *et al.,* 2021). Overall, five of these individuals were vaccinated with the BNT162b2 vaccine, with the remaining individuals having received the mRNA-1273 vaccination. There was a report of one separate case study where a 42-year-old woman who went into full remission after being diagnosed and treated for lupus nephritis class V in 2016, had relapsed a week after the first dose of the BNT162b2 vaccine. The patient went on to develop nephrotic syndrome with hyperlipoproteinemia and hypalbuminaemia ( Tuschen *et al.,* 2021). This data brings about the question as to whether there is a link between being vaccinated with an COVID-19 mRNA vaccine and the glomerulonephritis response and whether the COVID-19 mRNA vaccines are completely safe for use in certain vulnerable populations.

**Table 1.** Onset of IgAN in individuals after having the COVID-19 mRNA vaccines administered.

Patient	Age/Gender/race	Vaccine type	Previous IgAN history?	Onset of gross haematuria after first dose	Onset of gross haematuria after second dose	Comments	Reference
1	41yrs/F	BNT162b2	Yes	Day 2	Did not receive second dose	N/A	( Perrin *et al.,* 2021)
2	27yrs/F	BNT162b2	Yes	N/A	Day 2	N/A	( Perrin *et al.,* 2021)
3	52yrs/F/Asian	BNT162b2	Yes	N/A	Within 24hrs	N/A	( Rahim, Lin and Wang, 2021)
4	41yrs/F/Chinese	BNT162b2	No	N/A	Within 24hrs	Underlying gestational diabetes prior to vaccine. Undiagnosed IgAN revealed upon investigation	( Tan *et al.,* 2021)
5	60yrs/F/Malay	BNT162b2	No	N/A	Within 24hrs	Patient diagnosed with Hyperlipidaemia prior to any vaccination. Crescentic glomerulonephritis revealed upon investigation.	( Tan *et al.,* 2021)
6	22yrs/M	mRNA-1273	Yes	Day 2 and Day 25	Day 2	N/A	( Perrin *et al.,* 2021)
7	F	mRNA-1273	No	N/A	Within 2 weeks	Crescentic glomerulonephritis revealed upon investigation.	( Sacker, Kung and Andeen, 2021)
8	38yrs/F/White	mRNA-1273	Yes	N/A	< 24hrs	N/A	( Negrea and Rovin, 2021)
9	38yrs/F/White	mRNA-1273	Yes	N/A	< 24hrs	N/A	( Negrea and Rovin, 2021)

## Discussion

This study set out to establish the efficacy of the COVID-19 mRNA vaccines in DPs and KTRs. The seroconversion and cellular response were assessed in 15,072 participants after either two or three doses of an mRNA vaccine. The vaccines that were included in this analysis include BNT162b2 and mRNA-1273, with ChAdOx-1 nCoV-19 being excluded due to lack of available data.

Of the 9,913 DPs that were assessed for seroconversion after two doses of an mRNA vaccination, 85.29% showed positive seroconversion, with 14.71% of participants displaying no response. Comparatively, in the 4,822 KTRs that were assessed, the response was deemed ineffective when compared to DPs. 41.06% of KTRs displayed positive seroconversion, with the remaining 58.94% of participants showing no significant response to the vaccinations after two doses. We also assessed the seroconversion response after three doses of an mRNA vaccine. Of 1,513 participants, 76% of patients showed positive seroconversion with the remaining 24% of patients showing no response. When assessing cellular response in DPs and KTRs after two doses, 978 participants were included in the analysis, with a positive cellular response being observed in 61.86% of patients. The remaining 38.14% of participants showed no or little cellular response.

The BNT162b2 and mRNA-1273 vaccines both demonstrated a ~95% and ~94.1% efficacy at preventing COVID-19 in the general population respectively, during phase III clinical trials ( ClinicalTrials.gov number: NCT04470427; ClinicalTrials.gov number: NCT04368728) ( Polack *et al.,* 2020; Baden *et al.,* 2021). Comparatively, the seroconversion was also reported to be ~95% - 100% in healthy controls when fully vaccinated with either of the mRNA vaccines in four separate studies ( Stumpf *et al.,* 2021b; Ben-Dov *et al.,* 2022b; Grupper *et al.,* 2021; Sattler *et al.,* 2021). Our findings have highlighted a reduced seroconversion rate when compared to healthy controls of 85.29% and 41.06% in DPs and KTRs, respectively, albeit a better response in DPs when compared to KTRs alone. This could be due to several factors that should be taken into consideration. For example, age may play a factor in individual response to the vaccinations, with some studies showing younger KTRs may respond better to vaccination ( Broseta *et al.,* 2021; Torreggiani *et al.,* 2021). As well as this, it has also been highlighted that immunosuppression can impair immunity to COVID-19, with post-vaccination humoral response being inhibited by immunosuppressive therapy in KTRs ( Rincon-Arevalo *et al.,* 2021; Danthu *et al.,* 2021; Rozen-Zvi *et al.,* 2021; Deepak *et al.,* 2021; Strengert *et al.,* 2021). It is interesting to note that being fully vaccinated with BNT162b2 instead of mRNA-1273 was associated with a reduced immune response and yielded lower antibody titres in DPs and KTRs across several studies ( Affeldt *et al.,* 2021; Ionita *et al.,* 2022; Haller *et al.,* 2022; Broseta *et al.,* 2021; Stumpf *et al.,* 2021a).

This study also assessed whether a third dose of an mRNA vaccine generates an immune response in DPs and KTRs and whether this is stronger than the immune response observed after two doses. For this analysis, both DPs and KTRs were combined with 76% of the overall population favouring seroconversion after three doses compared to an average of 63.18% of DPs and KTRs favouring seroconversion after two doses. This strongly suggests an increase in the number of patients that display an immune response after a third dose of an mRNA vaccine. Further to this, there have also been some reports where it is noted that the immune response after two doses of an mRNA vaccine has declined, when reassessed at a later time point ( Boedecker-Lips *et al.,* 2022; Agur *et al.,* 2021; Dulovic *et al.,* 2022). From this, and since there appears to be an increase in the number of patients displaying an immune response in both DPs and KTRs after three doses, and no significant adverse side effects were reported, we do recommend administering a third dose to both population types.

As well as assessing seroconversion in DPs and KTRs, this study also looked at the cellular response upon being double vaccinated with an mRNA vaccine. Albeit a limited number of studies available, we found that 61.86% of 978 DPs and KTRs included in this assessment displayed a cellular response after two doses of an mRNA vaccine. This compares to ~97% - 100% of healthy controls displaying a positive cellular response in some studies ( Espi *et al.,* 2021; Strengert *et al.,* 2021; Sattler *et al.,* 2021; Bertrand *et al.,* 2021b). There has also been reports of an overall weaker T-cell response in DPs and especially KTRs compared to healthy controls ( Bertrand *et al.,* 2021a; Espi *et al.,* 2021; Strengert *et al.,* 2021; Sattler *et al.,* 2021). T-cell immunity is considered essential for long-lasting protection against infection, with reports of T-cell immunity being elicited after up to 17 years in patients who had recovered from Severe Acute Respiratory Syndrome (SARS) ( Le Bert *et al.,* 2020). Given that seroconversion or the antibody response can begin to wane overtime ( Boedecker-Lips *et al.,* 2022; Dulovic *et al.,* 2022; Agur *et al.,* 2021), eliciting a poor T-cell response can be associated with a worse prognosis for COVID-19 patients ( Mann *et al.,* 2020). With the production of broadly reactive T-cells, an individual may be able to develop better, long-term immunity, and the ability to better recognise potential variants in the future ( Peng *et al.,* 2020). Further work is now being undertaken to establish a mechanism to trigger broadly reactive T-helper cells and killer T-cells for an overall greater protective immunity to COVID-19 ( Dolgin, 2022; Heitmann *et al.,* 2022).

Separately, in the cohort that developed a glomerulonephritis response, albeit intriguing, it cannot be confirmed that the vaccination alone has triggered this response ( Klomjit *et al.,* 2021). Despite this, patients 1, 2, 3, 6, 8, and 9 had previously diagnosed IgAN with patients 4 and 5 having underlying medical health conditions, including gestational diabetes and hyperlipidaemia respectively. Upon investigation, both latter patients revealed previously undiagnosed IgAN and crescentic glomerulonephritis alongside patient 7, who also had a clear medical history revealing crescentic glomerulonephritis after examination. Further to this, patient 10 is the only case study with a relapse in lupus nephritis class V and II being reported, however, this further brings about the question as to whether the mRNA COVID-19 vaccines trigger a relapse in immune-mediated disease. Since it cannot be confirmed whether these relapses in disease are completely co-incidental due to the limited evidence highlighting this possible relationship, we recommend caution when administering COVID-19 mRNA vaccines in cases where glomerulonephritis may be present, with further investigation also being required for conclusive data.

It is important to note that there were certain limitations to this study. Firstly, the type of immunoassay used to evaluate seroconversion and cellular response was not standardised and thus, there may be some variation between studies and thresholds that were used to determine a positive test. It should also be considered that we were only able to assess whether there was an immune response rather than whether the immune response was sustained at different time points. In addition to this, across all the studies that met the inclusion criteria of this analysis, the type of reactive antibody measured to assess seroconversion was not standardised due to the variation between studies. Thus, upon evaluating the rate of seroconversion and antibody response after vaccination administration, we deemed it appropriate to group together the different antibody responses including IgG, IgA and IgM antibodies. One study also evaluated the number of detectable SARS-CoV-2-specific and receptor-binding domain (RBD)-specific antibodies ( Tai *et al.,* 2020). For similar reasons, the cellular response was also evaluated in a similar manner with the grouping of responses from different T-cell types, including CD4 ^+^ and CD8 ^+^ T cells. Further to this, although we had tried to limit our analysis to infection-naïve participants only, this was not always possible since some studies did not specify or clarify this information. There was also a great degree of variability between studies on the timing between doses and the timing between the last dose given and the assessment of the patients’ immune response to the vaccination. As a result, all studies were included, regardless of when the doses were given, and assessment was performed.

From the analysis performed in this study, there appears to be some publication bias and significant heterogeneity amongst studies. It is important to note that systematic heterogeneity is inevitable in this type of analysis due to the nature of the studies being investigated. Both publication bias and heterogeneity, as well as the limitations reported above must be taken into consideration when interpreting the results reported in this study. Overall, we have still been able to highlight the efficacy of the BNT162b2 and mRNA-1273 vaccines in DPs and KTRs. In conclusion, our analysis has highlighted that the mRNA vaccines yield an immune response in fewer CKD patients than when compared to healthy controls, albeit a better immune response in DPs than when compared to KTRs. Further to this, a third dose appears to be well-tolerated in both DPs and KTRs, and thus we recommend a third dose being administered to this population type to boost and ensure longevity in the patient’s immune response.

## Data availability

### Underlying data

All data underlying the results are available as part of the article and no additional source data are required.

## Reporting guidelines

Open Science Framework: PRISMA checklist for ‘A systematic review assessing the effectiveness of COVID-19 mRNA vaccines in chronic kidney disease (CKD) individuals’. https://doi.org/10.17605/OSF.IO/63PJX ( Goggolidou, 2022).

Data are available under the terms of the Creative Commons Zero “No rights reserved” data waiver (CC0 1.0 Public domain dedication).
