# Supplementary material for: A systematic review assessing the effectiveness of COVID-19 mRNA vaccines in chronic kidney disease (CKD) individuals
Source: F1000Res. 2022 Nov 22;11:909. Originally published 2022 Aug 8. [Version 2] doi: 10.12688/f1000research.122820.2 (PMC9732501; doi:10.12688/f1000research.122820.2)
Supplement: Peer review history

# Peer review: Reviewer response for version 2

**DOI:** 10.5256/f1000research.141313.r156324

**Published:** 2022-12-08

**Authors:** Marios Papasotiriou

**Affiliations:** 1 Department of Nephrology and Kidney Transplantation, University Hospital of Patras, Patras, Greece

Authors have responded adequately in their revised manuscript.

Are the rationale for, and objectives of, the Systematic Review clearly stated?

Yes

Is the statistical analysis and its interpretation appropriate?

Yes

Are sufficient details of the methods and analysis provided to allow replication by others?

Yes

Are the conclusions drawn adequately supported by the results presented in the review?

Yes

Reviewer Expertise:

Nephrology

I confirm that I have read this submission and believe that I have an appropriate level of expertise to confirm that it is of an acceptable scientific standard.

# Peer review: Reviewer response for version 2

**DOI:** 10.5256/f1000research.141313.r156323

**Published:** 2022-11-29

**Authors:** Katherine Karakoula

**Affiliations:** 1 School of Pharmacy, University of Wolverhampton, Wolverhampton, UK

I thank the authors for their time to address my comments appropriately.

 I also understand their response on the limited number of studies available to enable them to perform statistical analysis for the detection of positive seroconversion for DPs and KTRs after a third dose of an mRNA vaccine separately. 

 The paper is well-presented with sound evidence from their results to support their paper.

Are the rationale for, and objectives of, the Systematic Review clearly stated?

Yes

Is the statistical analysis and its interpretation appropriate?

Yes

Are sufficient details of the methods and analysis provided to allow replication by others?

Yes

Are the conclusions drawn adequately supported by the results presented in the review?

Yes

Reviewer Expertise:

Cancer genetics, immunological studies

I confirm that I have read this submission and believe that I have an appropriate level of expertise to confirm that it is of an acceptable scientific standard.

# Peer review: Reviewer response for version 1

**DOI:** 10.5256/f1000research.134859.r151580

**Published:** 2022-10-10

**Authors:** Katherine Karakoula

**Affiliations:** 1 School of Pharmacy, University of Wolverhampton, Wolverhampton, UK

The authors present a thorough systematic review on the effectiveness of mRNA vaccines for COVID-19 infection in DPs and KTRs patients.

 Overall, the review is well-presented with a detailed description of methods used for the analysis and good presentation of Figures. The review is highly cited with up-to-date references. The statistics used for the analysis are appropriate for systematic reviews and with sufficient details.

 The results are presented nicely albeit some additional information would be beneficial for this review. The authors investigated the efficacy of the 2 COVID-19 mRNA vaccines in DPs and KTRs by analysis of the seroconversion and cellular response in patients after either 2 or 3 doses of an mRNA vaccine. The authors found that a third dose of mRNA vaccine increased seroconversion by 76% in both DPs and KTRs together compared to an average of 63.18% after two doses. It would be helpful if the authors could do a separate analysis per set of patients (DPs and KTRs alone) after administration of a third dose of mRNA vaccine rather than grouping them together and then compared each set with the ones after a second vaccine dose. I believe the results of this analysis would help to understand better the requirement or not of a third dose of vaccine in these groups of patients.

 The discussion and conclusions support the results of this review.

Are the rationale for, and objectives of, the Systematic Review clearly stated?

Yes

Is the statistical analysis and its interpretation appropriate?

Yes

Are sufficient details of the methods and analysis provided to allow replication by others?

Yes

Are the conclusions drawn adequately supported by the results presented in the review?

Yes

Reviewer Expertise:

Cancer genetics, immunological studies

I confirm that I have read this submission and believe that I have an appropriate level of expertise to confirm that it is of an acceptable scientific standard, however I have significant reservations, as outlined above.

# Response

**Published:** 2022-11-18

**Authors:** Paraskevi Goggolidou

**Affiliations:** University of Wolverhampton, UK

We thank the reviewer for their comments. We have taken their points on board and edits to the initial manuscript can be seen as tracked changes. Please note that unfortunately, there are a limited number of studies available  and thus, to perform statistical analysis, the percentage of patients displaying positive seroconversion for both DPs and KTRs after a third dose were grouped together. Despite this, our data shows that separately, 89.13% and 61.29% of DPs and KTRs displayed positive seroconversion after a third dose of an mRNA vaccine, respectively.

# Peer review: Reviewer response for version 1

**DOI:** 10.5256/f1000research.134859.r149323

**Published:** 2022-09-08

**Authors:** Marios Papasotiriou

**Affiliations:** 1 Department of Nephrology and Kidney Transplantation, University Hospital of Patras, Patras, Greece

In this systematic review the authors assessed the response rate and overall effectiveness of vaccination against Covid-19 with mRNA vaccines in patients on dialysis or patients after kidney transplantation.

 The work overall is clearly presented and the most contemporary literature is cited. The design meets the standards of systematic reviews and analysis details are provided clearly in methods. The statistical analysis is appropriately performed, nevertheless some results need clarification. Firstly, it is stated that 17 studies with 1,513 participants were included to determine the effectiveness of 3 doses of the mRNA vaccines (either BNT162b2 or mRNA-1273) in both DPs and KTRs. In my opinion the seroconversion results after a third mRNA vaccine dose should be presented separately for DPs and KTRs. Moreover, it should be noted whether those patients, either DPs or KTRs, who did not show seroconversion with the 2 ^nd^ dose, had a substantial increase in antibody production after the 3 ^rd^ dose.

 In general, although the authors describe in detail the seroconversion ratio of DPs and KTRs after Covid-19 vaccination with an mRNA vaccine, they do not discuss about the actual protective threshold of antibodies that is reached in these groups of patients. Thus, I would propose to mention apart from seroconversion ratio, the ratio of patients that have a titer that is actually protective against severe Covid-19 infection as well.

 Finally, the source data are reported to be available and the final conclusions of the study are sound and are supported from the original results.

Are the rationale for, and objectives of, the Systematic Review clearly stated?

Yes

Is the statistical analysis and its interpretation appropriate?

Yes

Are sufficient details of the methods and analysis provided to allow replication by others?

Yes

Are the conclusions drawn adequately supported by the results presented in the review?

Yes

Reviewer Expertise:

Nephrology

I confirm that I have read this submission and believe that I have an appropriate level of expertise to confirm that it is of an acceptable scientific standard, however I have significant reservations, as outlined above.

# Response

**Published:** 2022-11-18

**Authors:** Paraskevi Goggolidou

**Affiliations:** University of Wolverhampton, UK

We thank the reviewer for their comments. In response to them, we have submitted a revised version of the article, with any amendments shown as tracked changes. Please note:

 1) There were a limited number of studies available and thus, to perform statistical analysis, the percentage of patients displaying positive seroconversion for both DPs and KTRs after a third dose were grouped together. Despite this, our data shows that separately, 89.13% and 61.29% of DPs and KTRs displayed positive seroconversion after a third dose of an mRNA vaccine, respectively.

 2) It was not confirmed in the studies whether antibody production/seroconversion after a third dose was compared to unvaccinated individuals or individuals who had received a second dose. Thus, in this study, we were unable to conclude whether there was an increase in antibody production after the third dose in those who did not show positive seroconversion after the second dose.

 3)Not all studies published the antibody titre levels, therefore it was not possible to conclude what the exact protective threshold was in DPs and KTRs vaccinated against severe COVID-19. Nonetheless, we have provided in Table 1 the threshold over which an individual's test is scored as seroconverted.

 It is important to note that it is not possible to deem immunity levels or identify protective thresholds from antibody testing alone. Further to this, we can confirm there is no specific antibody titre for protection against severe COVID-19. Accordingly, the U.S. Food and Drug Administration (FDA) states that “antibody testing is not currently recommended to assess immunity after COVID-19 vaccination” because a result from currently authorized SARS-CoV-2 antibody tests is not an indication of a specific level of immunity or protection from SARS-CoV-2 infection after the person has received a COVID-19 vaccination.
